# Gaze Following Is Modulated by Expectations Regarding Others’ Action Goals

**DOI:** 10.1371/journal.pone.0143614

**Published:** 2015-11-25

**Authors:** Jairo Perez-Osorio, Hermann J. Müller, Eva Wiese, Agnieszka Wykowska

**Affiliations:** 1 Department of Psychology, Ludwig-Maximilians University, Munich, Germany; 2 Graduate School of Systemic Neurosciences, Department Biology II Neurobiology, Ludwig Maximilian University, Planegg-Martinsried, Germany; 3 Department of Psychological Sciences, Birkbeck College, University of London, London, United Kingdom; 4 Department of Psychology, George Mason University, Fairfax, United States of America; VU University Amsterdam, NETHERLANDS

## Abstract

Humans attend to social cues in order to understand and predict others’ behavior. Facial expressions and gaze direction provide valuable information to infer others’ mental states and intentions. The present study examined the mechanism of gaze following in the context of participants’ expectations about successive action steps of an observed actor. We embedded a gaze-cueing manipulation within an action scenario consisting of a sequence of naturalistic photographs. Gaze-induced orienting of attention (gaze following) was analyzed with respect to whether the gaze behavior of the observed actor was in line or not with the action-related expectations of participants (i.e., whether the actor gazed at an object that was congruent or incongruent with an overarching action goal). In Experiment 1, participants followed the gaze of the observed agent, though the gaze-cueing effect was larger when the actor looked at an action-congruent object relative to an incongruent object. Experiment 2 examined whether the pattern of effects observed in Experiment 1 was due to covert, rather than overt, attentional orienting, by requiring participants to maintain eye fixation throughout the sequence of critical photographs (corroborated by monitoring eye movements). The essential pattern of results of Experiment 1 was replicated, with the gaze-cueing effect being completely eliminated when the observed agent gazed at an action-incongruent object. Thus, our findings show that covert gaze following can be modulated by expectations that humans hold regarding successive steps of the action performed by an observed agent.

## Introduction

Social interactions require the ability to predict and understand others’ behavior and its underlying intentions. To infer intentions and action goals, humans pick up various social signals, such as the others’ gestures or gaze direction, providing information about their focus of attention or intended action steps. There is ample evidence showing that humans attend to facial expressions and gaze direction of others (e.g., [1–4]). The capacity for discerning and following others’ gaze direction is an essential component of the ability to infer their current mental states, and helps establishing a common social context (e.g., [1], [5–9]). Gaze following has been extensively studied using the gaze-cueing paradigm (e.g. [10], [11]), in which a face, in canonical view, is typically presented centrally prior to the onset of a target in the periphery. Subsequently, the face’s eyes are directed towards one of the sides of the visual field–a potential target position. In a typical gaze-cueing study, processing of the target (detection, localization, or discrimination) is facilitated when the gaze direction and target position coincide, relative to when the gaze is directed elsewhere–the *gaze-cueing effect*. The gaze-cueing effect has been considered to rely on a reflexive mechanism (for review, see [12]), though more recently, it has been suggested that attentional orienting in response to gaze direction is susceptible to top-down modulation (e.g., [13–18]). For example, Teufel and colleagues ([19], [20]) proposed that information about others’ mental states influences automatic components of the gaze cueing effect. Similarly, [17] examined whether the mere belief that the observed agent is an intentional system influences gaze cueing. They manipulated the likelihood of adopting the intentional stance by instruction (in some conditions, participants were told that they were observing a human or a robot, in others, that they were observing a human-like mannequin or a robot whose eyes were controlled by a human). Interestingly, the authors found the magnitude of the gaze-cueing effect to be dependent on whether or not the gazer was construed as intentional, independently of the gazer’s physical appearance. Moreover, [18] analyzed the event-related potentials (ERPs) of the EEG signal recorded during the same task and found that the impact of beliefs about the gazer on the gaze-cueing effect was mirrored by a modulation of the target-locked P1 component at posterior-occipital electrode sites, indicating that already early processes of perceptual selection are prone to a top-down bias from higher-order cognition. Taken together, previous findings reveal that social perception is the result of an interactive process that involves the integration of bottom-up information provided by the stimulus and top-down influences by contextual variables.

If gaze direction provides important clues regarding the intentions of an observed agent, it is plausible that humans also use gaze direction to infer the subsequent (action) steps in complex action sequences, facilitating prediction of what others are going to do next and of crucial upcoming events in social interactions. Thus, arguably, observing others’ gaze behavior might elicit expectations about unfolding action sequences. Indeed, there is evidence that eye movements provide useful hints for understanding actions and predicting successive action steps: examining participants oculomotor behavior in a block-stacking task, [21] found that eye fixations invariably preceded proactively the landing points of manual movements during task execution. Importantly, Flanagan and Johansson observed similar eye movement patterns when participants merely observed an actor performing the same task. From this, they concluded that during action observation, humans implement similar oculomotor programs to those employed in action production. Similarly, [22] recorded eye movements of participants in natural situations, such as when making a sandwich. The results indicated that eye fixations predicted action steps: eye movements were strongly coupled to the task-relevant objects and preceded their use. The authors concluded that fixations serve to pick up critical information for performing the task and support high-precision movements. In summary, both studies reveal that gaze behavior provides good hints regarding successive action steps of others.

Similarly to gaze-induced expectations regarding successive action steps, humans also develop expectations regarding the way actions themselves unfold. For example, [23] showed that videos of action sequences incongruent with an action context produced longer recognition times, as compared to action sequences congruent with the context. Furthermore, several authors have claimed that observing actions triggers a corresponding action schema in the observer, including a goodness-of-fit evaluation between the observed action and the action schema [24]. An action schema can be described on two levels: the goal of the action (see, e.g., [25]) and its implementation, with the latter defined by the actor’s movements and the objects involved [26]. In sum, evidence suggests that people have expectations regarding subsequent action steps, as goal-directed actions follow a largely predefined pattern: a coherent sequence of steps, which makes actions relatively predictable [27]–a notion also supported by electrophysiological evidence [28].

The aim of the present study was to examine the interplay between expectations about an observed action and gaze-cueing effects. Consistent with the notion that, in daily life, gaze is informative with respect to subsequent action steps of an observed agent, and with empirical evidence in support of this notion [21, 22], we hypothesized that participants would have certain expectations regarding where an observed agent should gaze, given the action sequence the agent is performing. This, in turn, might affect gaze following (gaze-cueing effects), as gaze-cueing effects have been shown to be affected by how much ‘social sense’ is involved in the gaze behavior ([17], [18], [19], [29]). With regard to expected action sequences, if the observed gaze behavior is in line with the expected pattern, it would make more social sense to the observer–who might therefore more readily follow the other’s gaze relative to when the observed gaze behavior contravene expectations. To implement these ideas in an experimental study, we designed a paradigm in which a gaze-cueing protocol was embedded in a scenario that would evoke expectations regarding action sequences and gaze behavior of an observed agent. We were interested in examining how attention would be guided by gaze direction (gaze-cueing effects) when the expectations regarding action sequences would be either confirmed or violated. In our paradigm, a gaze-cueing procedure was embedded in a series of naturalistic photographs depicting a person (a woman named ‘April’) completing a goal-oriented task. At the beginning of each trial, an image introduced an action goal: it depicted either a guest asking her to bring her something to drink, or her flat mate asking her to fetch fabric softener to do the laundry. Afterwards, April was depicted in the kitchen with two bottles located to her left and right, respectively–each containing one of the liquids: either orange juice or fabric softener. Beside each bottle, there was a plastic cup. Subsequently, April gazed at either the action-congruent or action-incongruent bottle (e.g., in the context of bringing a drink to her friend, the congruent bottle would be the one with the orange juice, while the incongruent one would be that containing pink softener). In the final frame, some of the liquid (either orange juice or softener) appeared in one of the plastic cups (the target), and participants’ task was to discriminate whether the level of liquid in the plastic cup was high or low. Only one cup contained liquid and this always corresponded to the adjacent bottle. The crucial question was whether (liquid-level) discrimination performance would depends on whether the target was, or was not, gazed-at by the observed agent (*validity* of the gaze with respect to subsequent target presentation–the classical *gaze-cueing* manipulation) and how the validity effect would be modulated by whether the observed agent’s gaze was directed to the object congruent or incongruent with the action context (*congruency* of the actor’s gaze with respect to the action). We expected performance of the discrimination task to be affected by gaze validity, that is, to show the typical gaze-cueing effect. Importantly, we additionally hypothesized that the gaze-cueing effect would be modulated by whether the observed agent directed her gaze to an action-congruent or an action-incongruent object, in accordance with the ideas sketched above.

## Experiment 1

### Method

#### Participants

To determine the sufficient sample size for Experiment 1, we conducted an a-priori power analysis for the effect of congruency on gaze cueing, using: (i) a moderate effect size (*d*_*z*_ = .6), (ii) an α-error equal to .05, and (iii) a power level of .80 (as recommended by [30]). This analysis yielded an adequate sample size of 24. A total of 27 participants were recruited for the experiment to obtain 24 useable data sets (three of the initial 27 had to be excluded due to error rates higher than 15%). All 24 participants included in the analyses (age range 21–35 years, M = 24.36 years; 16 women; all right-handed) reported normal or corrected-to-normal vision, and normal color vision. None of the participants had previously taken part in an experiment with a similar design.

#### Ethical statement

**Fig 1:**
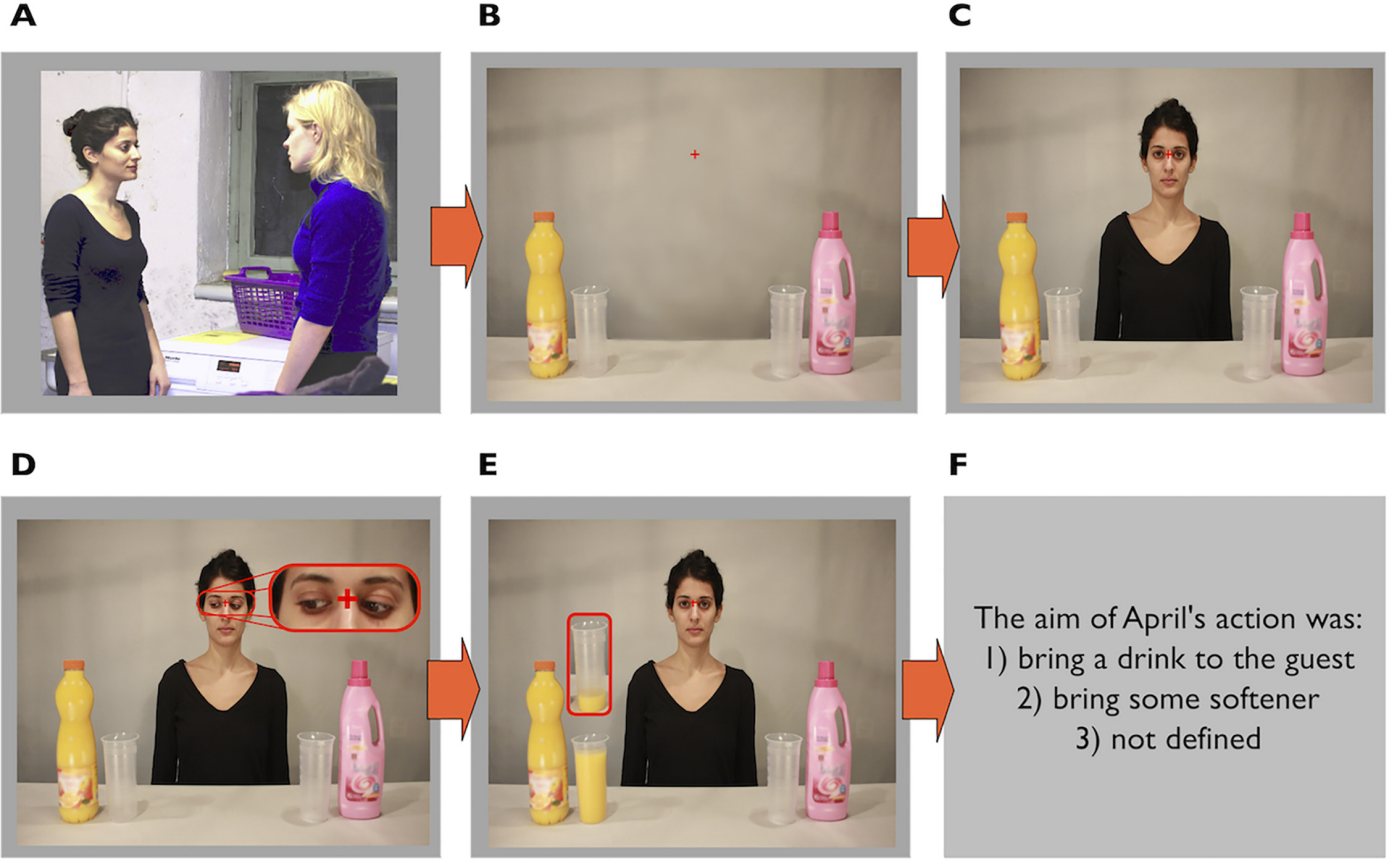
Schematic representation of an example trial in Experiment 1, depicting a ‘laundry’ context with an incongruent gaze direction and a validly cued target. Gaze direction in Frame D is zoomed-in only for the purpose of illustration. Frame E shows both the target with a low level of liquid as well as the target with high level of liquid. These targets are presented together only for illustration purposes; in the experiment proper, only one of the two targets was presented.

**Fig 2:**
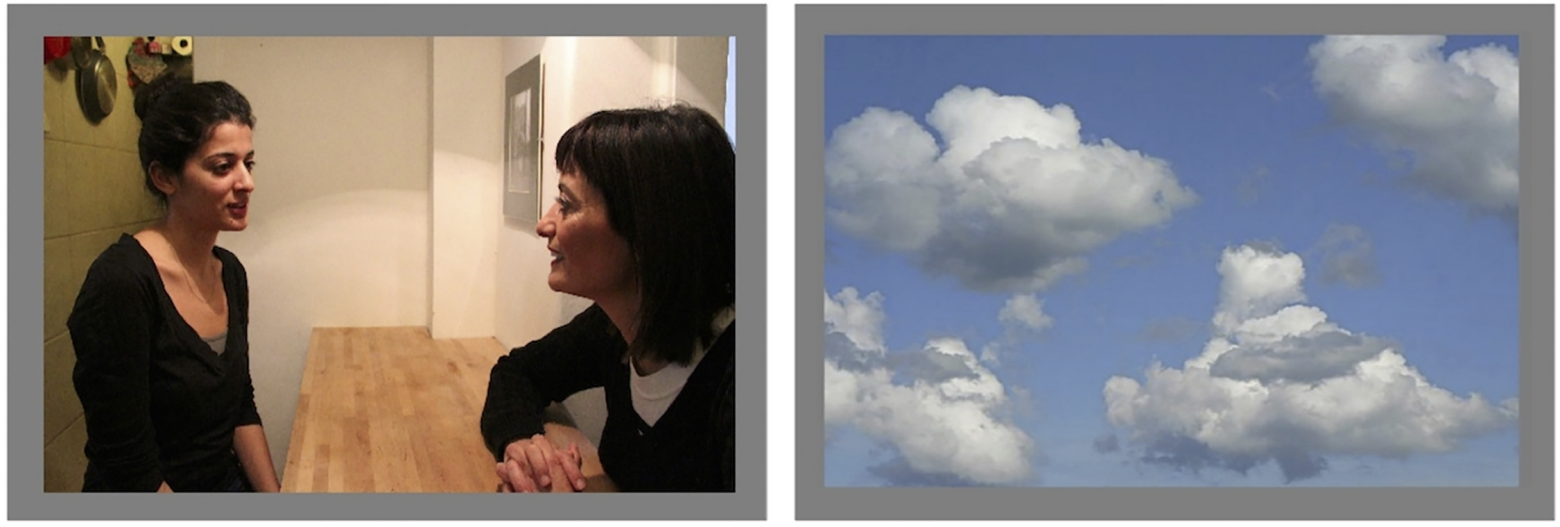
The two other action context images. The ‘drink’ context (left) and the ‘neutral’ context (right).

Experiment 1 (as well as Experiment 2; see below) was conducted at the Department of Experimental Psychology, LMU Munich, where all experimental procedures involving the collection of purely behavioral data (e.g., reaction times and error rates) with healthy adult participants (i.e., procedures that do not involve any invasive or potentially dangerous methods) are approved by the Department’s ethics committee in accordance with the Code of Ethics of the World Medical Association (Declaration of Helsinki). Data were stored and analyzed anonymously. Participants gave their informed, prior consent and were either paid or received course credit for participating. Finally, the individuals depicted in the photographic images in this article (cf. Figs 1, 2 and 4) gave written consent (in conformity with the PLoS ONE guidelines) for this material to be published.

### Apparatus and Materials

Participants performed the task seated in a dimly lit experimental cabin; looking at a 17” standard CRT monitor (100-Hz refresh rate, 1024 x 768 pixels screen resolution) positioned approximately 85 cm from their eyes. Stimulus presentation on the CRT was controlled by a Pentium IV PC using the E-Prime software (Psychology Software Tools, Pittsburgh, USA). The stimuli consisted of a series of color photographs taken for the purposes of this study–see Fig 1. The photographic images covered a screen area of 13.75° (width) x 10.35° (height) of visual angle; images were presented centrally, 6.7° from the screen borders.

#### Procedure and design

At the beginning of the experiment, participants received written instructions describing that a woman, April, would find herself in one of two situations: either a guest asks for something to drink, or a person who is living with her asks her to fetch fabric softener to do the laundry (Fig 1). Sometimes, there was no social situation and the context image was replaced by a picture of the sky with clouds, which meant that there was no specific task to be performed by April (Fig 2). Next, April goes to the kitchen (not explicitly presented in the trial sequence) where both potentially action-relevant items are to be found. Accordingly, the next image presented on the trial depicts a ‘kitchen-counter’ scene with two bottles, one positioned on the left and one on the right side, with a plastic cup next to each; one of the bottles contains orange juice (yellow), the other fabric softener (pink). Subsequently, the next image shows April standing between the bottles, gazing straight-ahead (in the direction of the observer). The following image shows April either making a gaze shift towards one of the bottles or maintaining straight-ahead gaze direction. In the final image, one of the cups (target) positioned next to one of the bottles appeared already containing a certain level of liquid–implying that April, in the meantime, had poured liquid into it. The sequence of actually lifting the bottle and pouring the liquid was not shown, in order to prevent the introduction of additional directional cues (arm extension, body posture) over and above April’s gaze direction. The participants’ task was then to determine whether the level of liquid (in the target cup) was either low or high (target discrimination task).

The most important manipulation was that before the frame containing the target (Fig 1D) the actor’s gaze was averted to either the bottle congruent or the bottle incongruent with the action context. That is, her gaze could be directed to the orange juice (yellow) in the ‘bring-a-drink’ scenario, or to the softener (pink) in the ‘laundry’ scenario (congruent conditions); or her gaze could be directed to the softener in the ‘drink’ context, or orange juice in the ‘laundry’ context (incongruent conditions). In the *congruency-neutral condition*, the image presented at the start of the trial depicted a sky with clouds, rather than a social scene (see Fig 2). Therefore, although the actor's gaze was directed to one of the bottles, this had no relation to an action context (because there was no action context specified in the congruency-neutral condition). This condition was introduced as a baseline for the gaze-cueing effects. All three gaze-congruency conditions were distributed equally across the experiment.

Additionally, the gaze was either valid or invalid with respect to the target position (Fig 1E). That is, the gaze direction could either coincide with the position at which the target would subsequently appear (valid trials) or not coincide (invalid trials). In the *neutral-validity* condition, the actor’s gaze remained looking straight ahead. The neutral validity condition was introduced to test whether compatibility of the *target* itself with respect to the action scenario had an impact on performance (independently of gaze direction). All three validity conditions we distributed equally across the experiment.

All nine conditions were pseudo-randomized across trials; also, the side on which each bottle was presented, the target type (orange juice or softener), and the level of liquid (low or high) were pseudo-randomized across trials–yielding a total of 48 trials per condition. The total number of trials was (9 x 48 =) 432, presented in 6 blocks of 72 trials each; an experimental session, including training, took some 80 minutes to complete.

Individual trials consisted of the following sequence of images (cf. Fig 1): First, a fixation point appeared at the center of the screen for 1000 ms (Fig 1A) Next, a centered context picture was presented together with an explanatory sentence (i.e., “Could you bring me a drink, please?” or “Could you fetch some softener, please?”) for 2500 ms; there were different images for the “drink”, “softener”, and “neutral” contexts–see Fig 2. (Fig 1B) This was followed by a picture displaying a kitchen counter with two bottles on it: yellow orange juice and pink softener, on opposite sides, equidistant from the center (5.71°); an empty transparent plastic cup (“glass”) was located next to each bottle; this picture remained on the screen for 600 ms (Fig 1C) April appeared between the two bottles looking straight ahead for 2000 ms (Fig 1D) For another 600 ms, she looked to one of the sides, or she kept looking straight ahead (neutral validity trials). (Fig 1E) An image was displayed (until response) in which April was presented again looking straight ahead, and which contained the response-relevant target: one of the glasses was partially filled with one of the two types of liquid to a high or a low level. Participants were asked to respond as fast and as accurately as possible by pressing the left mouse key for a low level and the right button for a high level of liquid. Finally, after the target response, an action context question was presented (Fig 1F) with three possible response options: ‘the aim of April’s action was: bring a drink to the guest, bring some softener, not defined’. We introduced the action context question in 2/3 of trials to ensure that the participants correctly encoded and kept in mind the context throughout the trial. The response was given by pressing the 1, 2, or 3 key on a standard computer keyboard, with accuracy (rather than speed) being stressed. The location of the response alternatives was randomized for each trial. Feedback regarding accuracy was given (1000 ms) right after the action context response: the word “correct” or “incorrect” in the center of the screen. Consecutive trials were separated by an inter-trial interval of 500 ms. Feedback about accuracy and reaction time in the target discrimination task for each entire block was provided in the breaks between blocks. Participants were asked to fixate in the middle of each frame and not move their eyes. They were explicitly informed that the direction of April’s gaze was not predictive with regard the location of the target.

#### Analysis

Consistent with our hypotheses, our main analysis focused on reaction times (RTs) in the target discrimination task as a function of gaze validity (valid, invalid) and gaze congruency with respect to action context (congruent, incongruent, neutral). RTs, measured as the time between target appearance and key press, were analyzed as follows: First, trials on which the action context question had not been queried were excluded from analysis, as well as trials on which the context probe was answered incorrectly (M = 6.66%, SD = 3.57). This was done to ensure that participants had actually attended the action scenario on the analyzed trials. Finally, trials with incorrect target responses (liquid-level discrimination) were eliminated (error rate M = 3.82%, SD = 2.64). Individual participants’ median RTs for each condition were calculated and subjected to a 3 × 2 repeated-measures ANOVA with the factors congruency (congruent/ incongruent/neutral) and validity (valid/invalid). In all analyses, degrees of freedom were adjusted according to Greenhouse-Geisser’s procedure when the sphericity assumption was violated.

### Results

Average median RTs and standard errors (in brackets) as well as error rates for each condition are presented in the Table 1 (see data in S1 Table for average per participant).

**Table 1:** Group average RTs and error rates Experiment 1.

Gaze Congruency	Validity			
	Valid		Invalid	
	RT	Error rate	RT	Error rate
Congruent	441 (15)	3.65%	480 (18)	4.25%
Incongruent	447 (15)	2.52%	462 (14)	3.91%
Neutral^a^	446 (14)	3.30%	464 (16)	3.73%

Group average of individual median RTs and associated standard errors of the means (SEMs, in ms), and a group average of error rates, as a function of cue validity and gaze congruency.

^a^‘Neutral’ refers to the ‘neutral congruency’ condition, in which observers were present with a neutral context image; in this condition, the observed gaze shift could still validly or invalidly cue the location of the target.

#### Reaction Times

**Fig 3:**
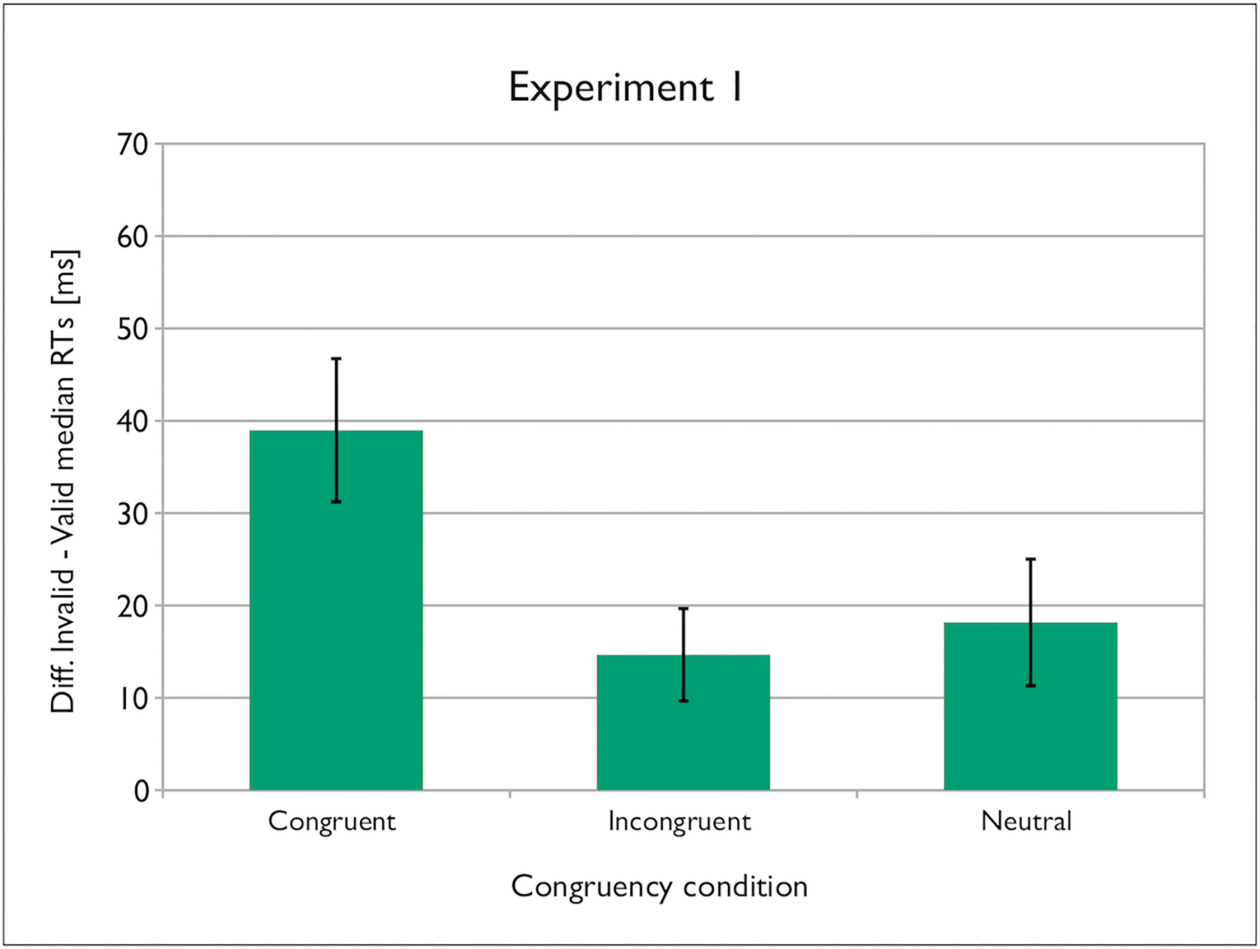
Gaze-cueing effect as a function of gaze congruency in Experiment 1. Error bars represent the confidence intervals (95% CIs) adapted for within-participants designs according to Cousineau’s [31] procedure.

The ANOVA on median RTs with the factors congruency (congruent, incongruent, neutral) and validity (valid, invalid) yielded a significant main effect of validity [*F* (1, 23) = 31.698, *p* = .00001, η_p_^2^ = .580]: discrimination RTs were shorter with valid (M = 444 ms; SEM = 14.6) than with invalid gaze cues (M = 468 ms; SEM = 15.94). The main effect of gaze congruency was not significant [*F* (2, 46) = .861, *p* = .429, η_p_^2^ = .036]. Importantly, the congruency × validity interaction was significant [*F* (2, 46) = 4.439, *p* = .017, η_p_^2^ = .162]. Although the validity effect was reliable in all three congruency conditions (valid vs. invalid for congruent gaze: ΔRT = 38.98 ms, *t* (23) = 5.028, 95% CI [22.94, 55.01], *p* = .00004, *d*_*z*_ = 1.02; for incongruent gaze: ΔRT = 14.66 ms, *t* (23) = 2.932, *p* = .007, 95% CI [4.32, 25.01], *d*_*z*_ = .60; and for neutral gaze: ΔRT = 18.16 ms, *t* (23) = 2.651, *p* = .014, 95% CI [3.99, 32.34], *d*_*z*_ = .54), it was larger in the congruent relative to the incongruent and the neutral condition. To assess the differences in gaze cueing effects as a function of gaze congruency, we calculated gaze-cueing effects (ΔRT = M RT_invalid_−M RT_valid_) and subjected them to planned comparisons across the three congruency conditions; see Fig 3.

**Fig 4:**
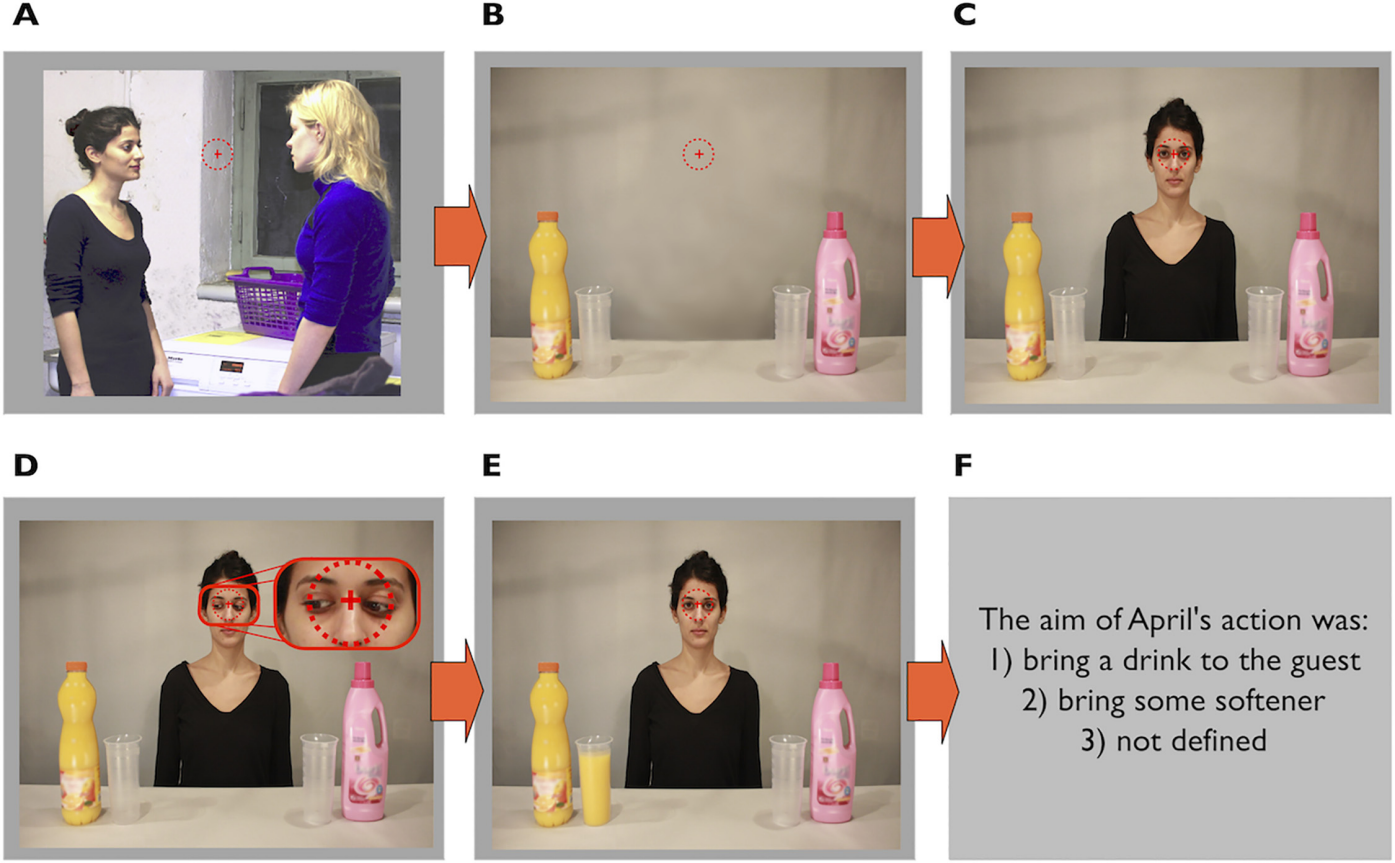
Schematic representation of an example trial in Experiment 2, with the same sequence of image frames as in Experiment 1 (Fig 1). The main difference between the experiments was that a fixation marker was presented throughout the image sequence. Participants’ eye movements were restricted to a circular area (represented by the dotted circle) of a radius of 2° around the (center of the) fixation cross.

The planned comparisons (two-tailed t-tests, Bonferroni-corrected) of the gaze-cueing effects revealed ΔRT to be significantly larger only for the congruent vs. incongruent conditions [*t* (23) = 3.153, *p* = .013, 95% CI [8.35, 40.26], *d*_*z*_ = .64], but not the congruent vs. neutral conditions [*t* (23) = 2.090, *p* = .144, 95% CI [.20, 41.41], *d*_*z*_ = .42]; the small difference between the neutral and incongruent conditions was not reliable [*t* (23) = .405, *p* = 1.00, 95% CI [-21.39, 14.39], *d*_*z*_ = .08], see Fig 3.

Note that we also examined whether compatibility of the target itself with the action context (e.g., orange juice in the ‘drinking’ scenario vs. orange juice in the ‘softener scenario’) influenced performance. For this purpose, we analyzed separately trials on which April’s gaze remained straight-ahead. We compared the target-compatible-, incompatible-, and neutral-context conditions for gaze-neutral (straight-ahead) trials. The ANOVA yielded no significant target-compatibility effect [*F* (2, 46) = 1.587, *p* = .216, η_p_^2^ = .065]: compatible (M = 503 ms; SEM = 18.48), incompatible (M = 514 ms; SEM = 19.25), and neutral (M = 514 ms; SEM = 19.41) (see data in S2 Table for average per participant). That is, in the gaze-neutral condition, target discrimination responses were not significantly affected by the compatibility of the target with respect to the action context.

#### Error rates

Participants’ error rates in the experiment were low overall (M = 3.56%, SD = 3.62). An ANOVA conducted on the error rates (analogous in design to the RT ANOVA) revealed none of the effects to be significant, all *p*s > .1. Nevertheless, in order to examine for potential speed-accuracy trade-offs (SATOs), we carried out an ANOVA analogous to the analysis on median RTs using so-called ‘inverse efficiency scores’ (IES) [32, 33]. IE scores are calculated by dividing individual (in our case: median) RTs for a particular experimental condition by an index of response accuracy: RT/(1 –p(E)), where p(E) is the error probability. That is, the RT value is increased the more the lower the accuracy associated with responses in this condition, effectively correcting for a speed-accuracy trade-off. Thus, examining the IE scores was designed to establish whether the pattern of RT results would remain unchanged when correcting for response errors. This analysis revealed essentially the same pattern of effects as the RT ANOVA: a significant validity effect, *F* (2, 23) = 34.130, *p* < .001, η_p_^2^ = .597, and a marginally significant interaction, *F* (2, 46) = 3.172, *p* = .051, η_p_^2^ = .121. That is, the pattern of RT results is reasonably robust, holding up even if when taking SATO effects into account.

### Discussion

The aim of Experiment 1 was to examine whether gaze cueing is affected by expectations regarding others’ gaze behavior in complex action sequences. To this end, we embedded a gaze-cueing protocol within an action context. The question of interest was whether participants’ performance of a discrimination task would be affected by the actor gazing (or not gazing) at the location of the subsequently appearing target object (gaze-cueing effect); and if the gaze-cueing effect would be influenced by whether the actor’s gaze behavior was in line with participants’ expectations induced by the action context in which the actor was embedded. The results revealed a main effect of validity, indicating that participants followed the observed agent’s gaze even though it was uninformative with respect to the position of the target. This is consistent with the idea that attentional orienting to gaze direction cues relies, to some extent, on a reflexive mechanism (e.g., [12], [10]). Importantly, however, the gaze-cueing effects were significantly modulated by the congruency of the gaze shift with respect to the action context: the gaze-cueing effect was larger when the observed agent directed her gaze to the context-congruent object, as compared to the context-incongruent object. In other words, the congruency of the observed agent’s gaze direction with respect to the action context played a significant role in the extent to which the agent’s gaze was followed: when the agent’s gaze shift confirmed participants’ prior expectations as to the object she would gaze at (in accordance with the action context), her gaze was followed to a larger extent compared to the action-incongruent gaze-shift condition.

In sum, Experiment 1 indicates that expectations regarding gaze behavior with respect to action plans influence the degree to which the gaze of an observed actor is followed. However, Experiment 1 did not permit us to determine whether the observed effects were indeed due to covert attention, or rather to overt attention. That is, alternatively, the slower responses in the invalid conditions might simply be due to participants having made (overt) saccades to the side opposite to the target location (despite the instruction to maintain fixation), rather than being attributable to violation of expectations (as assumed in the interpretation). Given this, the aim of Experiment 2 was to decide this issue by replicating the results of Experiment 1 while monitoring participants’ eye movements.

## Experiment 2

Experiment 2 was designed to isolate the influence of gaze direction cues and expectations regarding the observed gaze behavior on *covert* attentional orienting in a naturalistic scene. The paradigm and procedure were essentially the same as in Experiment 1, with the addition of monitoring participants’ eye fixation during critical frames of the trials using eye tracking. Also, to reinforce the instruction and make it easier for participants to maintain fixation during presentation of the critical stimulus frames (Fig 4, Frames A-E), a fixation cross was presented and remained on screen throughout frames A-E (see Fig 4).

If participants’ gaze deviated from the fixation cross by more than ±2° of visual angle (Fig 4, red dotted circle), the trial was aborted and repeated at the end of the block. This ensured that any effects obtained in Experiment 2 would not be attributable to shifts of overt attention. Note that the monitoring of participants’ eye movements in a gaze-cueing paradigm embedded in a naturalistic action scenario is a novel feature. While studies of simple, non-naturalistic scenarios have shown that observing gaze shifts evokes both covert and overt orienting (e.g., [34–36]), to our knowledge, no studies on orienting of covert attention in response to gaze cues in complex natural scenes have monitored eye movements.

### Method

#### Participants

In order to determine the sufficient sample size for Experiment 2, we conducted an a-priori power analysis for the effect of congruency (congruent vs. incongruent) on the size of gaze cueing, based on: (a) the effect size of Experiment 1 (*d*_*z*_ = .64); (b) an α-error equal to .05; and (c) a recommended power level of .80. This analysis yielded an adequate sample size of 22 participants. Accordingly, 22 healthy volunteers took part in Experiment 2 (age range 21–31 years, M = 24.8 years; 18 women; all right-handed), receiving monetary compensation or course credits for their participation. All participants had normal or corrected-to-normal vision, reported normal color vision, and provided written consent regarding participation in the experiment. None of the participants had taken part in Experiment 1 or in any other experiment with a similar design. Note that only participants who were able to maintain fixation during practice session (see Procedure section below) were admitted to the experiment proper.

#### Apparatus and Procedure

The apparatus and procedure (as well as the design) were essentially the same as in Experiment 1. However, there were a number of differences relating to the monitoring of participants’ eye movements. Participants’ head position and thus their eye-to-screen distance was ‘stabilized’ by means of a desk-mounted chin-and-headrest device positioned 60 cm in front of the CRT monitor. Eye movements were recorded monocularly (right eye) using an Eyelink 1000 tower-mounted eye-tracking system (SR-research Inc.; sampling rate: 2000 Hz; monocular accuracy: 0.25°–0.5°; resolution: 0.01°RMS (root mean square), related to the absolute sensor performance, the smaller the better). The apparatus was controlled using PsychToolbox 3 ([37–39] based on Matlab 2008a. Stimulus presentation on the screen was controlled by an Apple Mac Mini 2.3 using the same Matlab software. The images presented covered a screen area of 23.53° (width) x 17.06° (height) of visual angle, and each image was presented centrally, 7.9° from the borders of the screen. Note that while the images were of the same screen size as in Experiment 1, their perceived size (in terms of degrees of visual angle) was larger in Experiment 2 owing to a reduction of the eye-to-screen distance (according to the EyeLink user manual, the camera and illuminator should be placed at a distance of 40 to 70 cm from the observer, with the ideal tracking distance being 50 to 55 cm; SR Research Ltd. Mississauga, Ontario, Canada, 2005–2008). In order to facilitate maintenance of fixation, the sentences that had been presented under the context image in Experiment 1 were eliminated in Experiment 2.

After receiving written instructions, participants performed two practice blocks without eye tracking, and two additional blocks with gaze monitored by the eye tracker. The eye tracker was calibrated using a 13-point calibration procedure, which was immediately followed by a validation procedure. Calibrations were accepted if the mean error was less than 1.5°. In the experiment proper, when participants’ gaze diverted from central fixation by more than 2°, the trial was aborted and repeated at the end of the block (a trial block was finished only once participants had correctly completed all 24 trials within a block). In all other respects, the procedure was similar to Experiment 1, except that (i) the neutral-gaze condition was dropped (after having confirmed that target congruency per se did not affect participants’ responses [Experiment 1], there was no need to include the neutral gaze condition in the design of Experiment 2; thus, including a neutral condition in Experiment 2 would not have yielded any benefits for the design); and (ii) all trials included a question at the end regarding the action context (rather than only 66% of trials, as in Experiment 1). This was done in order to ensure that participants would encode and maintain in memory the action context under conditions that were more demanding than in Experiment 1 (in Experiment 2, participants were instructed to maintain fixation on the fixation cross for an extended period of time, which can be considered an additional task). Finally, (iii) trials with the ‘drink’ and ‘laundry’ scenarios had a second probe question (presented after the action context probe) regarding the correctness of the liquid with respect to the action context: “Did April take the correct liquid?” (Answers: number key 1 = yes, number key 2 = no); this additional question was introduced to reinforce the relevance of the action context to the entire task.

All six conditions were pseudo-randomized across trials; also, the side on which each bottle was presented, the target type (orange juice or softener), and the level of liquid (low or high) were pseudo-randomized across trials–yielding a total of 48 trials per condition (as in Experiment 1). The total number of trials was (6 x 48 =) 288, presented in 12 blocks of 24 trials each; an experimental session, including training, took some 60 minutes to complete. Feedback about accuracy and reaction time in the target discrimination task for each entire block was provided in the breaks between blocks. Participants were asked to fixate in the middle of each frame and not move their eyes and were explicitly informed that the direction of April’s gaze was not predictive with regard the location of the target.

#### Analysis

The same preprocessing steps and criteria for exclusion of trials were used as in Experiment 1. All participants maintained error rates lower than 15% in the action-context responses (M = 4.53%, SD = 3.19%). Median RTs were calculated for each participant and each condition and were subjected to a 3 x 2 repeated-measures ANOVA with the factors gaze congruency (congruent, incongruent, neutral) and validity (valid, invalid).

### Results

Average median RTs and standard errors (in brackets) as well as error rates for each condition are presented in the Table 2 (see data in S3 Table for average per participant).

**Table 2:** Group average RTs and error rates Experiment 2.

Gaze Congruency	Validity			
	Valid		Invalid	
	RT	Error rate	RT	Error rate
Congruent	515 (24)	12.59%	574 (25)	12.97%
Incongruent	543 (23)	13.26%	554 (24)	13.35%
Neutral^a^	515 (19)	12.03%	543 (22)	12.03%

Group average of individual median RTs and associated standard errors of the means (SEMs, in ms), and a group average of error rates, as a function of cue validity and gaze congruency.

^a^‘Neutral’ refers to the ‘neutral congruency’ condition, in which observers were present with a neutral context image; in this condition, the observed gaze shift could still validly or invalidly cue the location of the target.

#### Reaction Times

The 3 x 2 repeated-measures ANOVA of the median RTs with the factors congruency (congruent, incongruent, neutral) and validity (valid, invalid) revealed both main effects to be significant. The congruency effect [*F* (2, 42) = 4.058, *p* = .024, η_p_^2^ = .162] was due to RTs being faster in the neutral condition (M = 529 ms, SEM = 20 ms) as compared to the congruent and incongruent conditions (M = 545 ms, SEM = 24.17 ms; and, M = 549 ms, SEM = 23 ms, respectively), though planned comparisons (two-tailed t-test) revealed only the difference between the neutral and incongruent conditions to be significant [*t*(21) = 2.964, *p* = .007, *d*_*z*_ = .63]. The effect of validity [*F* (1,21) = 28.307, *p* = .00003, η_p_^2^ = .574] was owing to RTs being faster with valid (M = 525 ms, SEM = 21.27 ms) than with invalid gaze cues (M = 557 ms, SEM = 23.17 ms).

**Fig 5:**
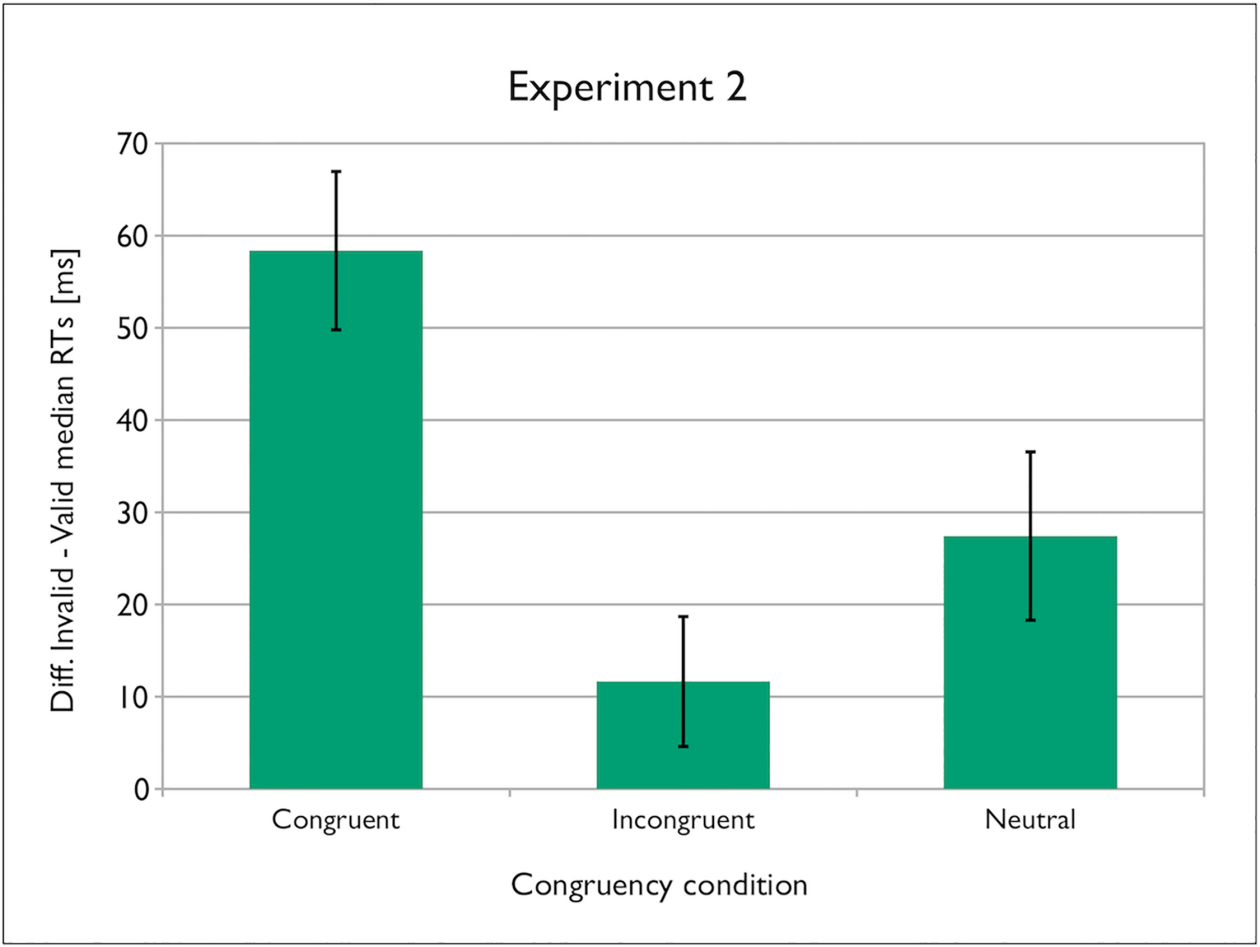
Gaze-cueing effect as a function of gaze congruency in Experiment 2. Error bars represent the confidence intervals (95% CIs) adapted for within-participants designs according to Cousineau’s [31] procedure.

Similarly to the Experiment 1, and importantly for the purposes of the study, the congruency x validity interaction was significant [*F* (2, 42) = 11.875, *p* = .000082, η_p_^2^ = .361], with the validity effect being significant only in the congruent and neutral gaze conditions, but not in the incongruent condition (valid vs. invalid for congruent gaze: ΔRT = 58.37 ms, *t* (21) = 6.803, *p* = .000001, 95% CI [40.53, 76.21], *d*_*z*_ = 1.44; for incongruent gaze: ΔRT = 11.65 ms, *t* (21) = 1.651, 95% CI [-3.03, 26.33], *p* = .114, *d*_*z*_ = 0.33; and for neutral gaze: ΔRT = 27.42 ms, *t* (21) = 3.001, *p* = .007, 95% CI [8.42, 46.43], *d*_*z*_ = .63). Planned comparisons (two-tailed t-test, Bonferroni-corrected) of the gaze-cueing effects (ΔRT = M RT_invalid_−M RT_valid_) revealed a significant difference in ΔRT between the congruent and incongruent gaze conditions [*t* (21) = 5.080, *p* = .00005, 95% CI [27.6, 65.84], *d*_*z*_ = 1.08] and between the congruent and neutral conditions, [*t* (21) = 3.272, *p* = .011, 95% CI [11.27, 50.61], *d*_*z*_ = 0.70], but no difference between the incongruent and neutral conditions [*t* (21) = 1.495, *p* = .450, 95% CI [-6.17, 37.73], *d*_*z*_ = .32]. In other words, the gaze-cueing effect was enhanced in the gaze-congruent condition as compared to the gaze-incongruent and gaze-neutral conditions (see Fig 5).

#### Error rates

An ANOVA conducted on the error rates (analogous in design to the RT ANOVAs) revealed no significant effects (*p*s > .75, except for the main effect of congruency). Only the main effect of congruency approached significance [*F* (2, 42) = 2.870, *p* = .068, η_p_^2^ = .120], with error rates being slightly larger for the incongruent (M = 13.3%, SEM = 3%) as compared to the congruent and neutral conditions (M = 12.78%, SEM = 2.9% and M = 12.1%, SEM = 2.8%, respectively). Post-hoc comparisons (two-tailed t-test, Bonferroni corrected) revealed no significant difference between the three congruency conditions (*p*s > .118). Note that the congruency effect on the error rates is of the same direction, and thus reinforces, the effect obtained in the RTs; that is, it is not indicative of a speed-accuracy trade-off. Similarly to Experiment 1, we conducted an ANOVA on inverse efficiency scores (IES) to account for potential speed-accuracy trade-off (SATO). The analysis showed an identical pattern of results as those obtained with uncorrected median RTs: a significant validity effect, *F* (1, 21) = 23.481, *p* < .001, η_p_^2^ = .528; a significant congruency effect, *F* (1.528, 32.095) = 7.813, *p* = .004, η_p_^2^ = .271; and a significant interaction, *F* (2, 42) = 5.748, *p* = .006, η_p_^2^ = .215)–again confirming that the pattern of RT effects cannot be explained by SATO influences.

### Comparison across Experiments 1 and 2

To examine whether the congruency factor had a similar influence on the effects of gaze cueing in both experiments, we conducted a mixed-design ANOVA on the gaze-cueing effects with *congruency* as within-participants factor and *experiment* as between-participants factor. This ANOVA yielded a significant main effect of gaze congruency [*F* (2, 88) = 15.681, *p* = .000001, η_p_^2^ = .263], but no significant interaction between gaze congruency and experiment (*p* = .237). Post-hoc comparisons (Bonferroni corrected) revealed significant differences in cueing effects between both the congruent and incongruent (*p* = .000001) and the congruent and neutral conditions (*p* = .002). Furthermore, independent-samples t-tests (with the assumption of homogeneity of variances ensured via Levene’s *F* test: *p* = .972, *p* = .233, and *p* = .150 for the gaze-congruent, incongruent, and neutral conditions, respectively) revealed no significant differences in the gaze-cueing effects between experiments for any of the congruency conditions (*p* = .100, *p* = .726, and *p* = .417 for the gaze-congruent, incongruent, and neutral conditions respectively). Thus, the two experiments yielded same pattern of results regarding the congruency factor.

### Discussion

To examine whether the findings of Experiment 1 were due to covert attention (rather than eye movement artifacts), Experiment 2 used essentially the same design as Experiment 1, including however the monitoring of participants’ eye fixation (trials with eye position shifts deviating by more than 2° from the fixation marker were excluded). Importantly, Experiment 2 replicated the results of Experiment 1 in all critical respects (in fact, without there being any significant differences in the cueing effects between the two experiments; see Comparison across Experiments 1 and 2), thus ruling out that the effect pattern was owing to overt, oculomotor orienting responses to the observed gaze shift. As in Experiment 1, the main effect of gaze cueing was significant, even with participants fixating the fixation cross throughout the critical trial frames A-E (Fig 4). This verifies that participants covertly attended the object that the actor gazed at, even though the actor’s gaze direction was spatially uninformative with respect to the target object. Likewise, the modulation of the gaze-cueing effect by the congruency of the actor’s gaze (the gazed-at object) with the action context cannot be attributed to a congruency-dependent modulation of overt eye movements, but instead reflects a modulation of covert attentional orienting.

Importantly, no gaze-cueing effect was obtained in Experiment 2 when the actor’s gaze was incongruent with the action context. That is, the facilitation normally engendered by gaze cues was eliminated when expectations regarding which object would be gazed at (given the overarching action context) were violated. Furthermore, the congruent gaze condition yielded stronger gaze-cueing effects relative to the neutral action-context baseline. Restated, relative to the baseline, the gaze-cueing effect was actually enhanced when the actor’s gaze ‘complied’ with observers’ expectations regarding the object that would be gazed at in order to achieve the ultimate action goal.

## General Discussion

The present study was designed to examine–using a naturalistic scenario–how the gaze direction of an observed actor and the observer’s expectations, induced by the action context, would influence the gaze-cueing effects. The results of both experiments showed that task performance (i.e., discrimination of the liquid level in the target) depended on gaze-cue validity with respect to the target side, with attention following the gaze direction of the observed agent (‘April’)–the typical gaze-cueing effect. Importantly for the purposes of this study, the gaze-cueing effect was modulated by whether the actor had gazed at an action-congruent or at an incongruent object: specifically, the cueing effect was significantly enhanced when the actor gazed at an object congruent with the action context, relative to an incongruent object (Experiments 1 and 2), and it was entirely eliminated when the actor gazed at an object incongruent with the action context, relative to a congruent object (Experiment 2).

Consequently, gaze cueing appeared to be modulated by participants’ expectations with regard to the gaze behavior in the context of upcoming action steps within an overarching action sequence. This is in line with the notion that humans activate a certain action schema [26] when observing others in action and (possibly implicitly) expect the gaze of the observed agent to precede (and, thus, provide a pointer to) successive action steps [21],[22]. Hence, when the observed gaze behavior confirms participants’ expectations concerning the action sequence, the gaze cueing effects seem to be enhanced relative to when the gaze behavior violates the expectations. Moreover, gaze-cueing effects can even be entirely suppressed when the gaze behavior violates action-related expectations. The complete lack of a validity effect seen in Experiment 2 when the actor looked at an object that was action-incongruent further attests to the impact of top-down control on bottom-up-driven attentional orienting in response to gaze direction.

This set of findings is in line with a wide body of literature suggesting that higher-order cognitive mechanisms can modulate orienting of attention in response to spatial cues (e.g., [40]–[42]). Teufel et al. [19], Wiese et al. [17], and Wykowska et al. [18] extended this evidence to gaze following, demonstrating that gaze-cueing effects can be reduced [17] or even eliminated [18] by top-down regulation of the mechanisms involved. In particular, [17] found that the context in which a gaze shift is performed–in their study: the presence versus absence of physical reference objects to which the gaze would refer–modulates the degree to which attentional resources are deployed to the cued location. On this basis, they proposed that this modulation is mediated via a top-down mechanism which binds the gaze shift (in central vision) to a referred-to object (in peripheral vision). Subsequently, [29] extended the notion of ‘context’ to also include social factors, such as knowledge of the reliability of the cue provider, to account for their finding that gaze following was modulated by whether participants perceived the gaze behavior displayed by the observed gazer as reliable and highly predictive or not. Applied to the results of the present experiment, the top-down component can engender both an enhancement of the gaze-cueing effect (namely, when the actor’s gaze is seen to be shifted to the action-congruent object) and a suppression of the default, presumably ‘reflexive’, gaze following (when the actor’s gaze is shifted to an action-incongruent object).

Finally, the present results extend earlier findings in that they suggest a link between action prediction and gaze following. This is in accordance with neuroimaging results showing activation of the superior temporal sulcus (STS) during action prediction [43] and updating predictions after a violation of an expected action sequence [8]. Interestingly, the STS region is also involved in gaze-direction detection and gaze cueing [3]. Hence, also at the neural level, following gaze seems to be closely linked to anticipatory mechanisms in action observation. In more general terms, the close link between gaze following and action prediction seems to be very adaptive: In order to interact with others, we need to know what the others are going to do next [44]. As people tend to look at objects they are planning to manipulate, gaze direction is informative about the identity (*what*?) and spatial location of attended objects (*where*?). Together with knowledge about people’s preferences (*who*?), which can be acquired directly by interacting with them or indirectly by either observing someone interacting with another person or receiving information about a person, we make inferences about their internal states (*why*?) [44]–so as to predict which action is most likely to be performed next under the given circumstances. Thus, in our paradigm, knowing that April was asked to bring a glass of orange juice to a guest, one would predict that she would be looking for an empty cup and a bottle of orange juice (in order to pour some juice and bring the filled glass to the guest). Therefore, through linking processing of gaze direction with action prediction within a single paradigm, the current study demonstrates that spatial information derived from gaze direction and context information about the action goal can be combined in order to predict consecutive steps in an action sequence. Taken together, the present findings have implications concerning the actual function of the gaze-following mechanism and the role it plays in natural daily-life scenarios: arguably, gaze following has developed not just to pick up signals that others convey regarding potentially relevant events in the environment, but also, and conceivably foremost, to enable us to infer what others are going to do next [5],[44].

Concerning methodological implications, using a sequence of naturalistic photographs brought our paradigm closer to more ecologically valid, real-life social scenarios. Admittedly, though, it is still a rather artificial protocol in which participants are just observing a series of static images. Arguably, however, this step needed to be made between entirely artificial stimuli and completely realistic protocols. Having a sequence of images allowed us to maintain experimental control over factors of interest and to circumvent certain confounds (such as involuntary attentional capture or motion-related effects). With the present paradigm providing a first step into more naturalistic scenarios, future research should take the design even closer to real life (e.g., by using video or virtual-reality technology) and to a more interactive protocol (rather than merely involving an observational stance). We contend that when the protocol is made more naturalistic, the effects observed in the current study might turn out even stronger.

## Conclusion

The present study is, to our knowledge, the first to examine how attention is deployed within a naturalistic visual scene as a result of the gaze direction displayed by an observed actor and the observer’s expectations regarding the unfolding of the action sequence in a complex action scenario. With the use of a novel paradigm that uses naturalistic images, we show that the gaze-cueing effects, reflecting covert shifts of attention, can be modulated–either enhanced or (even entirely) suppressed–dependent on whether the gaze behavior of the observed agent (gazing at action-congruent or incongruent objects) does or does not fit with the expectations that participants hold with regard to the unfolding action sequence. In summary, our findings indicate that one of the key functions of gaze following is to monitor and predict others’ actions.

## Supporting Information

S1 Table Individual average of median RTs per condition Experiment 1. (PDF)

S2 Table Individual average of median RTs for neutral validity Experiment 1. (PDF)

S3 Table Individual average of median RTs per condition Experiment 2. (PDF)
