# Correction: Gaze Following Is Modulated by Expectations Regarding Others' Action Goals

**DOI:** 10.1371/journal.pone.0170852

**Published:** 2017-01-20

**Authors:** Jairo Perez-Osorio, Hermann J. Müller, Eva Wiese, Agnieszka Wykowska

The sixth sentence of the fourth paragraph in the General Discussion section should have cited Sebanz et al., 2009 instead of reference 44. The correct sentence should read: As people tend to look at objects they are planning to manipulate, gaze direction is informative about the identity (what?) and spatial location of attended objects (where?). Together with knowledge about people’s preferences (who?), which can be acquired directly by interacting with them or indirectly by either observing someone interacting with another person or receiving information about a person, we make inferences about their internal states (why?) (Sebanz et al., 2009)–so as to predict which action is most likely to be performed next under the given circumstances.

The correct reference is: Sebanz N, Knoblich, G. Prediction in Joint Action: What, When, and Where. Top Cogn Sci. 2009; 1:353–367. doi:10.1111/j.1756-8765.2009.01024.x
