# Supplementary material for: Gaze Following Is Modulated by Expectations Regarding Others’ Action Goals
Source: PLoS One. 2015 Nov 25;10(11):e0143614. doi: 10.1371/journal.pone.0143614 (PMC4659552; doi:10.1371/journal.pone.0143614)
Supplement: S1 Table — (PDF) [file pone.0143614.s001.pdf]

S1 Table. Individual average of median RTs per condition Experiment 1

Gaze Congruency	Congruent		Incongruent		Neutral	
Participant	Valid	Invalid	Valid	Invalid	Valid	Invalid
1	406,0	457,0	415,0	433,0	438,0	387,5
2	430,0	481,0	466,5	474,5	465,5	555,0
3	545,5	563,0	564,0	573,0	460,0	580,0
4	445,0	462,5	456,0	431,0	408,0	423,0
5	507,0	506,0	438,0	452,0	456,0	467,0
6	608,0	731,0	616,0	623,0	615,0	643,5
7	418,0	502,0	429,0	471,0	420,0	442,0
8	410,0	440,0	437,0	443,0	432,0	452,0
9	401,0	424,5	395,5	458,0	400,0	449,0
10	351,0	388,0	397,0	372,0	364,0	402,0
11	429,0	446,5	443,0	427,0	443,0	422,0
12	396,0	478,0	432,5	467,0	456,0	467,0
13	491,0	514,0	469,0	474,5	487,0	498,0
14	357,0	411,0	368,5	400,5	405,0	424,0
15	499,0	647,0	517,0	570,0	569,0	587,5
16	595,5	597,0	591,0	608,0	588,0	602,0
17	422,0	427,0	427,5	419,0	427,0	425,0
18	392,0	414,0	396,5	377,0	378,0	403,0
19	492,0	521,0	477,5	492,0	491,0	489,0
20	417,5	432,0	406,5	461,0	414,0	406,5
21	463,0	484,0	430,0	437,0	447,5	455,0
22	305,0	326,5	313,0	356,0	318,0	308,0
23	352,0	352,0	360,5	370,0	389,5	397,0
24	452,0	515,0	489,0	498,0	430,5	453,0
