# Supplementary material for: Gaze Following Is Modulated by Expectations Regarding Others’ Action Goals
Source: PLoS One. 2015 Nov 25;10(11):e0143614. doi: 10.1371/journal.pone.0143614 (PMC4659552; doi:10.1371/journal.pone.0143614)
Supplement: S2 Table — (PDF) [file pone.0143614.s002.pdf]

S2 Table. Individual average of median RTs for neutral validity Experiment 1

Target compatibility / Participant	Compatible	Incompatible	Neutral
1	428,0	423,5	458,0
2	569,0	546,0	546,0
3	650,5	594,5	633,0
4	497,0	499,0	505,0
5	468,5	553,5	564,0
6	744,0	804,0	806,5
7	509,0	527,5	508,5
8	465,0	479,0	477,0
9	495,0	500,0	485,0
10	414,5	457,0	420,0
11	556,0	515,0	529,5
12	517,5	521,0	536,0
13	533,0	541,0	524,5
14	429,0	444,0	402,0
15	530,0	557,0	566,5
16	609,0	624,0	636,0
17	489,5	417,0	470,0
18	443,5	427,0	460,0
19	599,0	598,5	602,0
20	435,0	448,0	424,0
21	518,5	539,0	505,5
22	327,0	340,0	338,0
23	369,0	396,0	426,0
24	496,0	587,0	515,0
