# Supplementary material for: Gaze Following Is Modulated by Expectations Regarding Others’ Action Goals
Source: PLoS One. 2015 Nov 25;10(11):e0143614. doi: 10.1371/journal.pone.0143614 (PMC4659552; doi:10.1371/journal.pone.0143614)
Supplement: S3 Table — (PDF) [file pone.0143614.s003.pdf]

S3 Table. Individual average of median RTs per condition Experiment 2

Gaze Congruency	Congruent		Incongruent		Neutral	
Participant	Valid	Invalid	Valid	Invalid	Valid	Invalid
1	720,2	751,0	726,6	764,9	638,8	699,4
2	408,4	442,1	406,6	396,8	413,8	387,9
3	551,1	742,3	564,3	637,4	567,2	711,1
4	604,8	695,5	686,9	655,7	640,4	674,7
5	514,5	530,0	517,0	533,2	482,9	472,0
6	776,0	793,5	730,8	726,0	642,5	690,2
7	462,6	545,0	504,1	498,7	488,2	487,5
8	453,1	519,5	497,7	422,4	400,3	448,2
9	454,6	466,9	452,3	460,2	471,1	462,2
10	446,4	464,3	496,6	477,2	417,5	476,0
11	558,1	619,9	603,3	640,1	558,7	594,5
12	658,3	741,4	696,0	661,4	631,2	647,1
13	467,0	531,0	506,7	551,9	531,6	507,1
14	501,3	551,5	505,2	517,2	508,6	539,1
15	533,9	577,6	586,1	605,9	629,8	607,9
16	485,1	531,3	474,6	500,6	467,1	485,4
17	423,6	444,9	415,6	416,1	407,3	450,9
18	682,2	719,7	707,0	731,9	607,5	684,6
19	409,6	484,0	492,9	526,5	519,8	468,1
20	347,4	428,4	395,2	416,9	398,8	429,2
21	506,7	613,9	546,8	601,5	505,1	562,6
22	380,7	436,0	427,9	454,2	404,8	450,5
